# Detailed operational data for three Norwegian houses equipped with wood stoves: Sub-Hourly room-level measurements of indoor environmental quality, space-heating power, stove surface temperature, and household total electric power

**DOI:** 10.1016/j.dib.2025.112411

**Published:** 2025-12-22

**Authors:** Abolfazl Mohammadabadi, Øyvind Skreiberg, Laurent Georges

**Affiliations:** a Department of Energy and Process Engineering, Varmeteknisk laboratorium Gløshaugen, Trondheim, 7034, Trøndelag, Norway; b SINTEF Energi AS, Kolbjørn Hejes vei 1 A, Trondheim, 7034, Norway

**Keywords:** Wood stove, Thermal comfort, Space heating, Electricity consumption, Electrical grid

## Abstract

Behind-the-meter data were collected in three residential buildings in Trondheim, Norway, heated with direct electric panels and a wood stove, as part of the SusWoodStoves research project, which aimed to investigate the influence of wood stove operation on electric power consumption. The dataset is characterized by high resolution in time, space (that is, room level), and the number of recorded physical quantities, allowing for a wide range of investigations related to indoor climate, energy use, and building–grid interaction. Specifically, the dataset includes indoor and outdoor environmental variables such as air temperature, CO_2_, TVOC, radon concentrations, and particulate matter; global solar irradiation has been recorded from a nearby meteorological station; electric power is measured for each heat emitter and on the main meter; and the surface temperature of the wood stove is recorded to monitor its operation. Measurements were sampled every 5 min, with periods ranging from two to six months.

Specifications Table

**Table:** 

Subject	Engineering & Materials science
Specific subject area	Indoor climate and energy use in buildings, building-grid interaction
Type of data	Table (csv files)
Data collection	Data were collected in late winter 2023 for the first house and during the winter of 2025 for the other two houses. All dwellings are located in Trondheim and are heated exclusively with electric panel heaters and a wood stove. The 2023 campaign served as a pilot study, during which the experimental setup was developed and validated. The methodology was finalized during this 2023 campaign so that the resulting dataset could be considered valid. The other two campaigns were conducted in 2025 and constituted the main data collection periods. Monitoring periods were classified into two categories: (i) periods in which occupants were allowed to use the wood stove and (ii) periods in which stove use was prohibited. Each period was designed to include days with both mild and cold outdoor conditions.
	A set of sensors and data-loggers has been placed in the dwellings:
	• Electricity consumption: The total building consumption was measured using a data logger connected to the smart meter (AMS) while plug-in power meters (smart plugs) were connected to each electric heating panel. • Outdoor environmental conditions: Outdoor air temperature, relative humidity, and CO_2_ concentration were collected from one sensor placed outside the houses in a shaded area. Global solar irradiation was measured by a weather station located on the Gløshaugen campus of NTNU in Trondheim. • Indoor climate measured by a dedicated sensor installed in each room. • Wood stove temperature measured using a surface temperature sensor placed directly on the wood stove.
Data source location	Country: Norway
	City: Trondheim
Data accessibility	Repository name: Mendeley Data
	Data identification number: 10.17632/8b7xwh2366.5
	Direct URL to data:https://data.mendeley.com/datasets/8b7xwh2366/5

## 1 Value of the Data

• The dataset is valuable, as it provides **high-resolution measurements** from three residential buildings heated with electric panels in Trondheim, Norway. It includes high-frequency time series data with a 5-min sampling interval, while the raw data were collected at intervals ranging from 10 s to 5 min. The dataset covers periods of two to four months. Measurements were collected in all rooms within buildings (multizone) and comprise room-level space-heating power, indoor temperature, CO_2_ concentration, radon, total volatile organic compounds (TVOCs), relative humidity (RH), air pressure, and particulate matter (PM), as well as outdoor environmental conditions (air temperature, RH, CO_2_ concentration, and global solar irradiation).
• All three houses are equipped with a **wood stove**, which was operated manually during the measurement period. The temperature of the stove’s external surface was recorded to monitor its operation and to support a better understanding and modeling of the relationship between stove use, indoor temperature, and electricity consumption.
• The data set is original. Several datasets with **sub-hourly behind-the-meter (BTM) data** have already been made publicly available, see e.g., [1], [2]. These dataset differ in the type of buildings (residential, office, educational buildings), their local climate, occupancy (i.e., occupied vs. unoccupied), and heating system. They also show variations between the physical variables collected, with indoor air quality parameters being reported only in a few studies [2]. Our dataset covers a case that has not been addressed in the past, complementing the existing datasets. It addresses small residential buildings with occupants, heated by electric panels and a wood stove, with room-based measurement, including space-heating power, indoor air temperature, and some key IAQ parameters. Given the limited thermal capacitance of electric panels, the electric power consumed is close to the space-heating power emitted to the room. This enables focusing on the heat balance of each thermal zone of the building in detail.
• The dataset can be used for different purposes. It can support the development and validation of **data-driven models** that relate indoor climate to space-heating power, for example, using grey-box modeling [3], [4]. These data-driven models of the building thermal dynamics are typically used as control-oriented models for advanced indoor climate and energy management applications, such as Model Predictive Control (MPC) [5]. The richness of the data further enables detailed modeling: CO_2_ and TVOC concentrations can serve as proxies for occupancy, while electricity use (excluding space-heating power) can be used as a proxy for internal heat gains in the building. In addition, researchers can employ this dataset to **quantify and model the effects of wood stove use** on space heating and electric power consumption. Finally, the dataset can be used to validate **disaggregation algorithms** that aim to evaluate the space-heating power of the entire household from its total electric consumption [6].

## 2 Objective

The original objective was to collect data from three residential buildings heated with electric panels to study the relationship between the indoor thermal environment, space-heating, and electric power consumption, as well as the operation of a wood stove. A key research question was to characterize the impact of wood stove operation on electric power consumption and the associated stress relief on the electricity grid. This work was conducted within the framework of the research project *Sustainable Wood Stoves through Stove, Building Integration and Value Chain Optimization* (SusWoodStoves). However, the high resolution of the dataset enables it to be used for a wide range of other purposes.

## 3 Data Description

### 3.1 Dataset 1: Pilot house

The pilot house is located in the western part of Trondheim, Norway. It is a detached building divided horizontally into two separate dwellings. The structure has a concrete foundation, wooden walls, and double-pane windows. The floors are finished with wooden parquet. The residence selected for measurements is naturally ventilated and comprises two floors plus a basement. The first floor includes a kitchen, bathroom, bedroom, and living room, while the second floor contains a bathroom and three bedrooms.

**Fig. 1:**
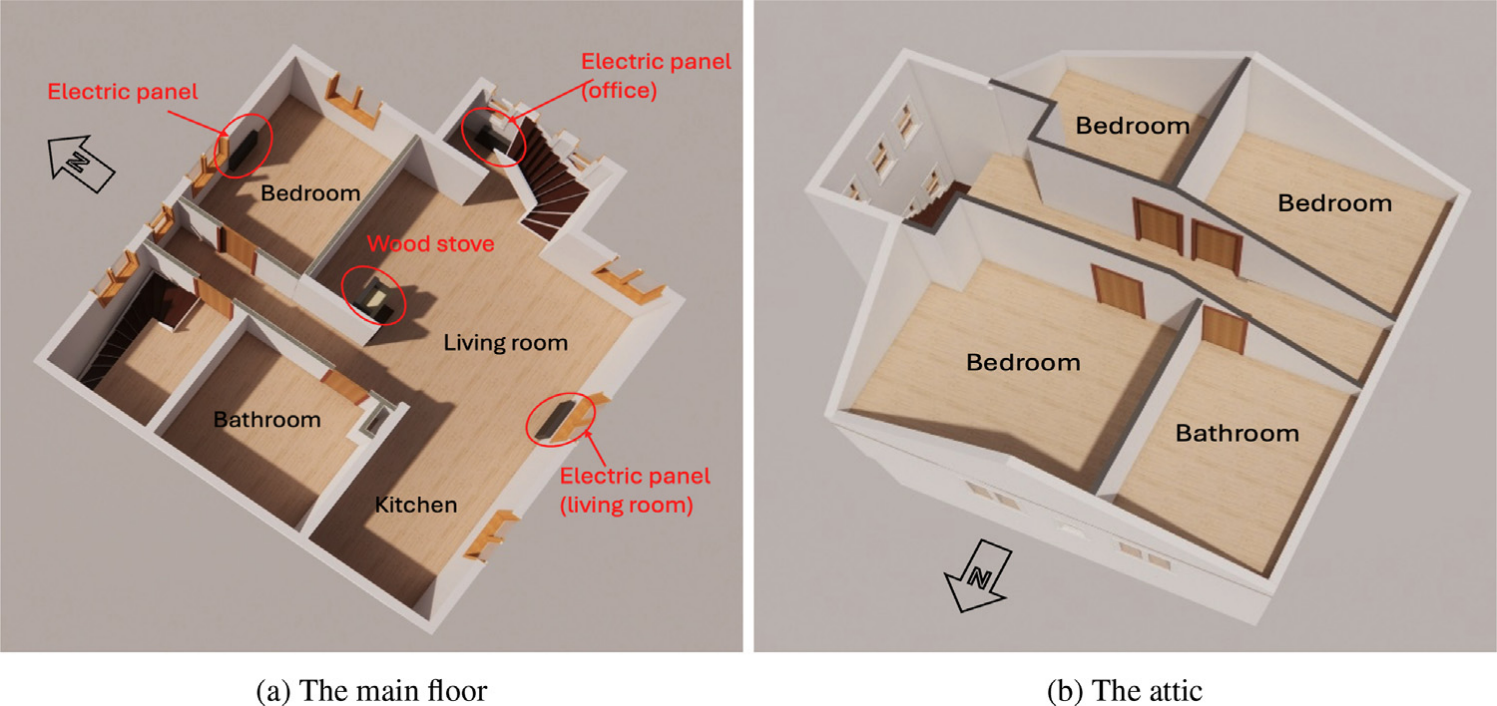
3D plan of the pilot house - electric panels and wood stove.

The first floor is heated by three 1kW electric radiators and a wood stove. The electric radiators operate with simple on/off control, maintaining a set-point temperature defined by the occupants. The wood stove is located in the living room, which also contains two of the electric radiators, while a third radiator is installed in a bedroom (Fig. 1).

Data (see Table 1) were collected during winter, from 28 February to 6 April 2023, and divided into two periods. From 28 February to 21 March, the occupants were allowed to use both the wood stove and the electric radiators for space heating. From 22 to 28 March, the wood stove was not operated and only electric radiators were used. The second period was shorter due to the approaching end of the heating season.

**Table 1:** Name of the Excel sheet, measured physical variables, and their descriptions for the Pilot House (dataset 1).

File name	Sheet name	Variable name	Unit	Description
Dataset_1	main_power	DT	-	UTC date-time, format: YYYY-MM-DD HH:MM:SS
		Pe	W	Smart meter readings averaged over five-minute intervals
	office_panel_1	DT	-	UTC date-time, format: YYYY-MM-DD HH:MM:SS
		Psh_off	W	Power of electric panel in office room averaged over five-minute intervals
	bedroom_panel_2	DT	-	UTC date-time, format: YYYY-MM-DD HH:MM:SS
		Psh_bed	W	Power of electric panel in bedroom averaged over five-minute intervals
	livingroom_panel_3	DT	-	UTC date-time, format: YYYY-MM-DD HH:MM:SS
		Psh_liv	W	Power of electric panel in living room averaged over five-minute intervals
	1bathroom_climate	DT	-	UTC date-time, format: YYYY-MM-DD HH:MM:SS
		Rn	$\text{Bq}/m^{3}$	Radon level
		CO_2_	ppm	CO_2_ level
		RH	%	Relative humidity
		T	${}^{\circ }$C	Air temperature
		TVOC	ppb	Total volatile organic compounds level
		p	mbar	Air pressure
		PM1	μg/m${}^{3}$	Particulate matter with a diameter of 1 μm or less
		PM2.5	μg/m${}^{3}$	Particulate matter with a diameter of 2.5 μm or less
	2hallway_climate	DT	-	UTC date-time, format: YYYY-MM-DD HH:MM:SS
	3bedroom_climate	Rn	$\text{Bq}/m^{3}$	Radon level
	4hallway_climate	CO_2_	ppm	CO_2_ level
	5kitchen_climate	RH	%	Relative humidity
	6bathroom_climate	T	${}^{\circ }$C	Air temperature
	7bedroom_climate	TVOC	ppb	Total volatile organic compounds level
	9bedroom_climate	p	mbar	Air pressure
	10livingroom_climate			
	11bedroom_climate			
	8outdoor_climate	DT	-	UTC date-time, format: YYYY-MM-DD HH:MM:SS
		Rn	$\text{Bq}/m^{3}$	Radon level
		CO2	ppm	CO2 level
		RH	%	Relative humidity
		T	${}^{\circ }$C	Air temperature
		TVOC	ppb	Total volatile organic compounds level
		p	mbar	Air pressure
		G	W/$m^{2}$	Average global radiation on a horizontal plane
	wood_stove1	DT	-	UTC date-time, format: YYYY-MM-DD HH:MM:SS
		T	${}^{\circ }$C	Air temperature behind the wood stove, near wall and floor
		RH	%	Relative humidity behind the wood stove
		light	lux	Light behind the wood stove
		Ts	${}^{\circ }$C	Surface temperature of the wood stove
	wood_stove2	DT	-	UTC date-time, format: YYYY-MM-DD HH:MM:SS
		T	${}^{\circ }$C	Air temperature behind the wood stove, near wall and floor
		RH	%	Relative humidity behind the wood stove
		light	lux	Light behind the wood stove
		Ts	${}^{\circ }$C	Flue pipe temperature of the wood stove

**Fig. 2:**
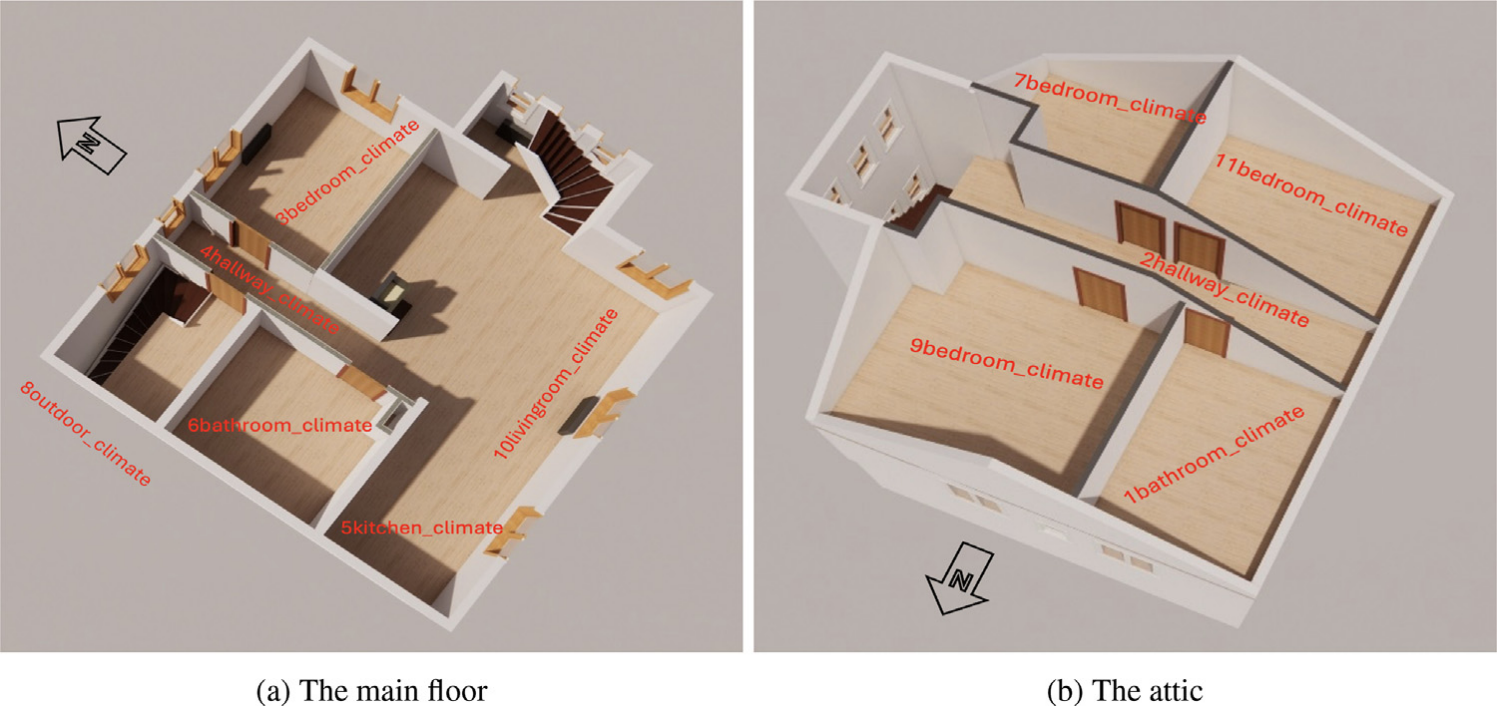
3D plan of the pilot house - climate sensors.

Ten indoor climate sensors were installed throughout the house, including in the bedrooms, bathrooms, kitchen, and living room (Fig. 2). Outdoor conditions were monitored using a single climate sensor. Two additional sensors measured the surface and flue temperatures of the wood stove (for further details, see [7]).

### 3.2 Dataset 2: Semi-detached house 1

**Fig. 3:**
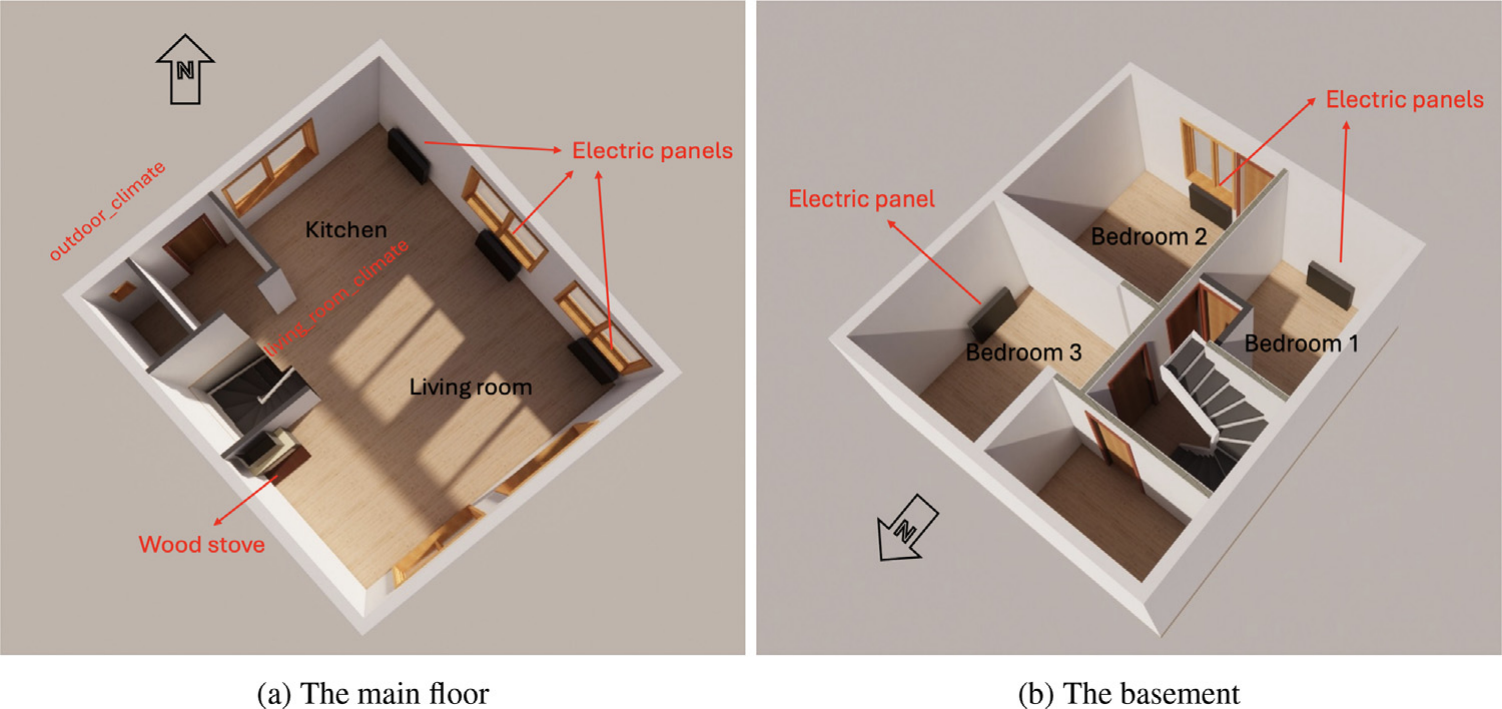
3D plan of the semi-detached house 1.

The studied building is a horizontally divided semi-detached wooden house located in the southwest of Trondheim. The house is approximately 25 years old, with a floor area of about 120 $m^{2}$. It is insulated with 15 cm of material in both walls and roof and is equipped with double-pane windows. The building relies on mechanically balanced ventilation and includes a basement level (−1 floor) where the bedrooms are located. The basement includes three bedrooms, each heated by an electric panel heater. On the first floor, the layout consists of a living room and kitchen with large windows, complemented by three additional electric panel heaters and a wood burning stove (Fig. 3). The household consists of a family of four with typical heating habits, primarily relying on electric panel heaters. During the 2024 measurement period, only the electric panels were used, whereas in 2025 the heating was supplemented with the wood stove for a period of about 10 weeks, from 23 January to 5 April (Table 2).

**Table 2:** Dataset 2, Excel sheets, variables, and descriptions for semi-detached house 1.

File name	Sheet	Variable	Unit	Description
Dataset_2	main_power	DT	–	UTC date-time, format: YYYY-MM-DD HH:MM:SS
		Pe	W	Smart meter readings averaged over five-minute intervals
	DHW	DT	–	UTC date-time, format: YYYY-MM-DD HH:MM:SS
		Pdhw	W	Domestic hot water power averaged over five-minute intervals
	electric_panel_1	DT	–	UTC date-time, format: YYYY-MM-DD HH:MM:SS
	(Bedroom 1)	Psh_1	W	Electric panel 1 power averaged over five-minute intervals
	electric_panel_2	DT	–	UTC date-time, format: YYYY-MM-DD HH:MM:SS
	(Bedroom 2)	Psh_2	W	Electric panel 2 power averaged over five-minute intervals
	electric_panel_3	DT	–	UTC date-time, format: YYYY-MM-DD HH:MM:SS
	(Living room)	Psh_3	W	Electric panel 3 power averaged over five-minute intervals
	electric_panel_4	DT	–	UTC date-time, format: YYYY-MM-DD HH:MM:SS
	(Living room)	Psh_4	W	Electric panel 4 power averaged over five-minute intervals
	electric_panel_5	DT	–	UTC date-time, format: YYYY-MM-DD HH:MM:SS
	(Bedroom 3)	Psh_5	W	Electric panel 5 power averaged over five-minute intervals
	electric_panel_6	DT	–	UTC date-time, format: YYYY-MM-DD HH:MM:SS
	(Living room)	Psh_6	W	Electric panel 6 power averaged over five-minute intervals
	living_room_climate	DT	–	UTC date-time, format: YYYY-MM-DD HH:MM:SS
	bedroom1_climate	Rn	$\text{Bq}/m^{3}$	Radon level
	bedroom2_climate	CO2	ppm	CO2 level
	bedroom3_climate	RH	%	Relative humidity
		T	${}^{\circ }$C	Air temperature
		TVOC	ppb	Total volatile organic compounds level
		p	mbar	Air pressure
	outdoor_climate	DT	–	UTC date-time, format: YYYY-MM-DD HH:MM:SS
		Rn	$\text{Bq}/m^{3}$	Radon level
		CO2	ppm	CO2 level
		RH	%	Relative humidity
		T	${}^{\circ }$C	Air temperature
		TVOC	ppb	Total volatile organic compounds level
		p	mbar	Air pressure
		G	W/$m^{2}$	Average global radiation on a horizontal plane
	wood_stove	DT	–	UTC date-time, format: YYYY-MM-DD HH:MM:SS
		T	${}^{\circ }$C	Air temperature behind the wood stove, near the wall and floor
		RH	%	Relative humidity behind the wood stove
		light	lux	Light behind the wood stove
		Ts	${}^{\circ }$C	Surface temperature of the wood stove

### 3.3 Dataset 3: Semi-detached house 2

The semi-detached house 2 is located in the southeast of Trondheim, Norway. It is a vertically divided house with a wooden foundation and wooden walls. The house is poorly insulated and relies on natural ventilation. It consists of two floors and a loft living room.

**Fig. 4:**
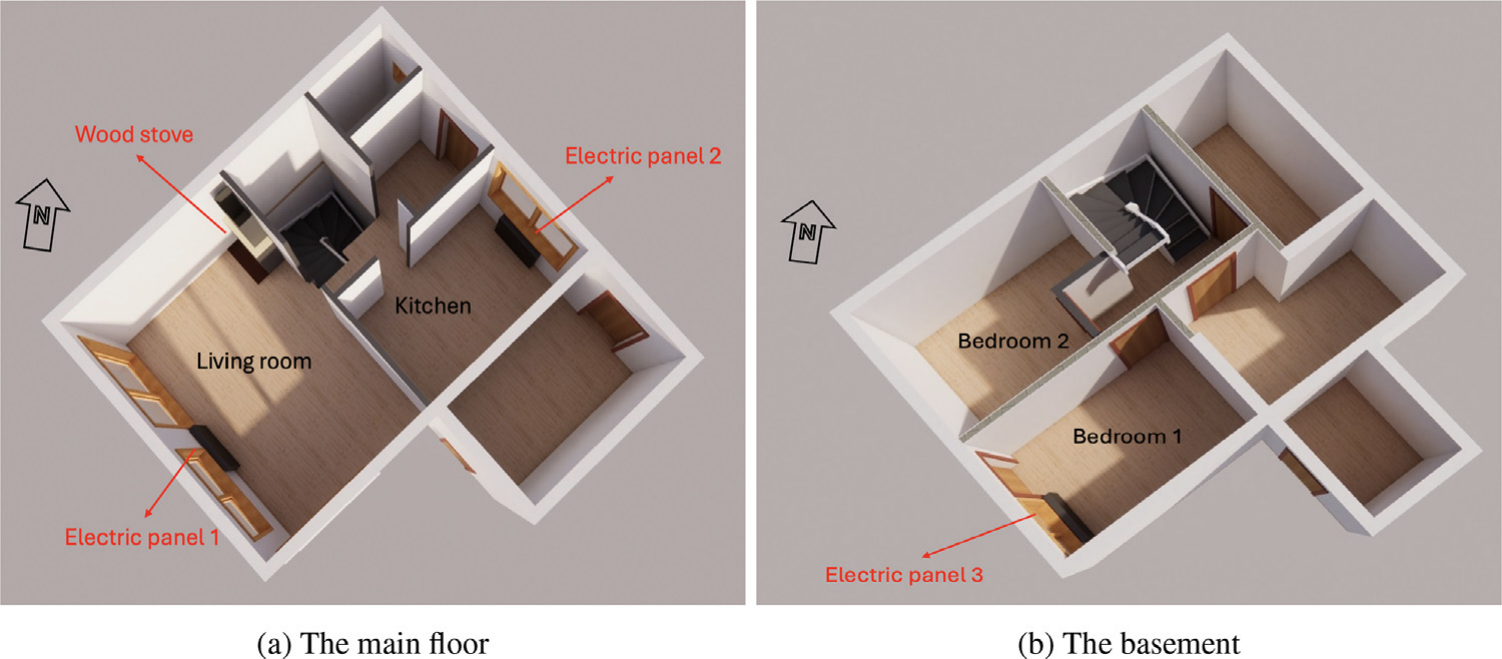
3D plan of the semi-detached house 2.

The first floor features a separate kitchen, a living room with large windows and a wood-burning stove, a bathroom with underfloor heating, and a laundry room. The basement includes two bedrooms and a separate bathroom. Heating is provided by electric panels in all rooms, except the bathroom, which uses floor heating (Fig. 4). Residents often use the wood-burning stove in winter.

The household consists of a family of four (two adults and two children) and exhibits typical Norwegian heating habits.

The measurement period was divided into two phases: with and without wood stove operation. At the beginning of the measurements, the wood stove was in use, while from February 5th onward the wood stove was no longer used, and heating was provided exclusively by the electric panels (Table 3).

**Table 3:** Dataset 3, Excel sheets, variables, and descriptions for semi-detached house 2.

File name	Sheet	Variable	Unit	Description
Dataset_3	main_power	DT	–	UTC date-time, format: YYYY-MM-DD HH:MM:SS
		Pe	W	Smart meter readings averaged over five-minute intervals
	electric_panel_1	DT	–	UTC date-time, format: YYYY-MM-DD HH:MM:SS
	(Living room)	Psh_1	W	Electric panel 1 power averaged over five-minute intervals
	electric_panel_2	DT	–	UTC date-time, format: YYYY-MM-DD HH:MM:SS
	(Kitchen)	Psh_2	W	Electric panel 2 power averaged over five-minute intervals
	electric_panel_3	DT	–	UTC date-time, format: YYYY-MM-DD HH:MM:SS
	(Bedroom)	Psh_3	W	Electric panel 3 power averaged over five-minute intervals
	living_room_climate1	DT	–	UTC date-time, format: YYYY-MM-DD HH:MM:SS
	bedroom1_climate	CO2	ppm	CO2 level
	bedroom2_climate	RH	%	Relative humidity
	kitchen_climate	T	${}^{\circ }$C	Air temperature
		TVOC	ppb	Total volatile organic compounds level
		p	mbar	Air pressure
	living_room_climate2	DT	–	UTC date-time, format: YYYY-MM-DD HH:MM:SS
		Rn	$\text{Bq}/m^{3}$	Radon level
		CO_2_	ppm	CO_2_ level
		RH	%	Relative humidity
		T	${}^{\circ }$C	Air temperature
		TVOC	ppb	Total volatile organic compounds level
		p	mbar	Air pressure
		PM1	μg/m${}^{3}$	Particulate matter with a diameter of 1 μm or less
		PM2.5	μg/m${}^{3}$	Particulate matter with a diameter of 2.5 μm or less
	outdoor_climate	DT	–	UTC date-time, format: YYYY-MM-DD HH:MM:SS
		Rn	$\text{Bq}/m^{3}$	Radon level
		CO2	ppm	CO2 level
		RH	%	Relative humidity
		T	${}^{\circ }$C	Air temperature
		TVOC	ppb	Total volatile organic compounds level
		p	mbar	Air pressure
		G	W/$m^{2}$	Average global radiation on a horizontal plane
	wood_stove	DT	–	UTC date-time, format: YYYY-MM-DD HH:MM:SS
		T	${}^{\circ }$C	Air temperature behind the wood stove, near the wall and floor
		RH	%	Relative humidity behind the wood stove
		light	lux	Light behind the wood stove
		Ts	${}^{\circ }$C	Surface temperature of the wood stove

It should be noted that the sensor livingroom_climate2, initially positioned close to the wood stove, recorded elevated temperature values of up to 45 ${}^{\circ }$ C before being relocated to a position further away from the stove. In addition, the power measurements for 2025 in the kitchen, living room, and bedroom are of less quality compared to the rest of the dataset, and caution is advised when using these values. The corresponding raw data are not available due to data loss on the Raspberry Pi. In addition, the bedroom power measurements were unintentionally turned off in 2024 and no data is available for that period.

**Fig. 5:**
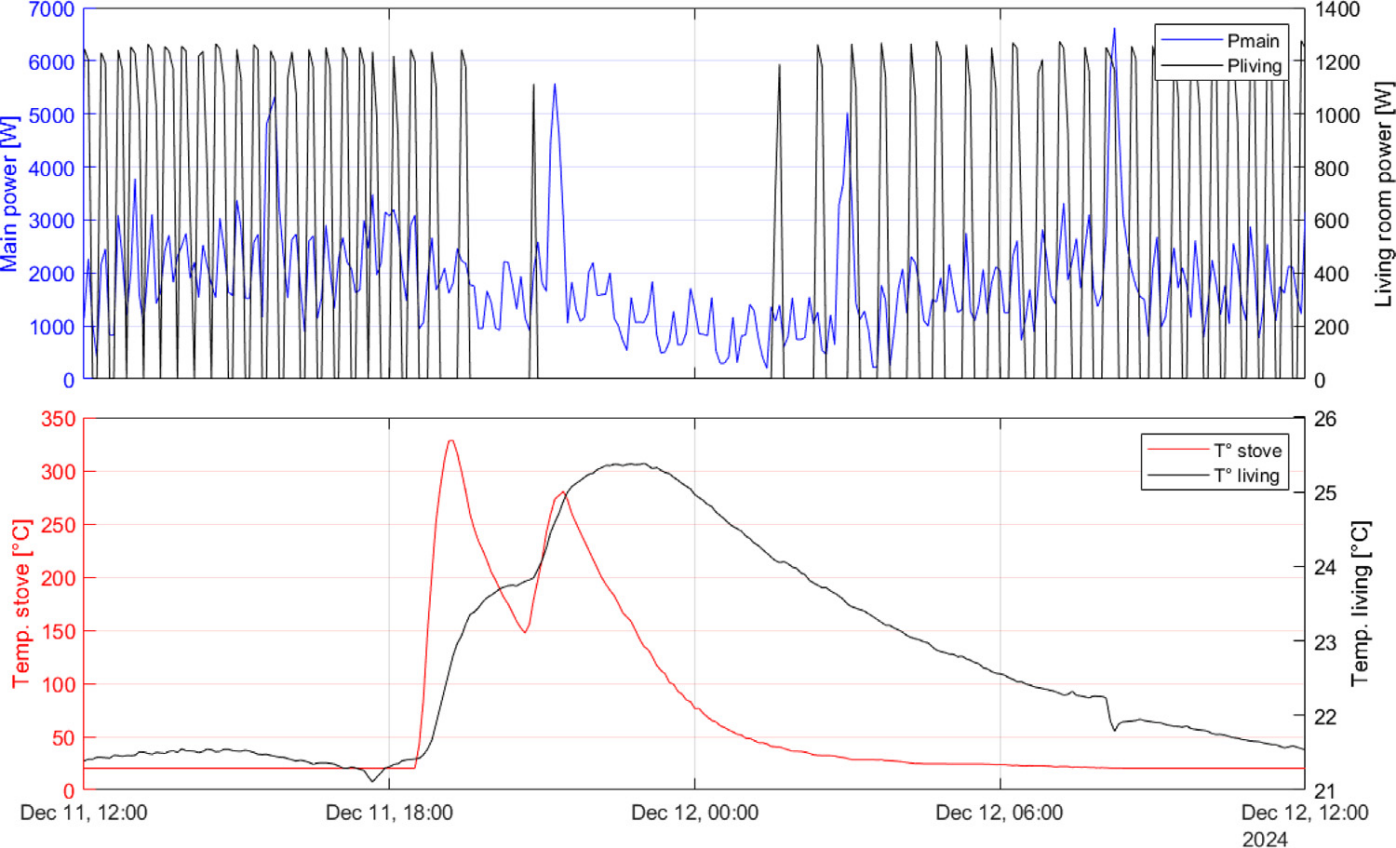
Overview of a subset of Dataset 3 showing the main building power, the space heating power of the living room, and the temperatures of the wood stove and the living room.

Fig. 5 presents an example from dataset 3 and illustrates the effect of wood stove temperature on both electric power use and indoor temperature. The stove was ignited at 18:00, resulting in a rapid increase in the measured surface temperature, reaching approximately 300 ${}^{\circ }$C. The surface temperature of the stove is not spatially uniform, and the contact between the sensor and the stove surface may introduce a small contact thermal resistance. Therefore, the recorded temperature should be interpreted as an indicator of the temporal evolution of the stove’s heat output rather than an exact magnitude.

The indoor temperature in the living room follows the stove surface temperature with a delay that reflects the thermal mass of the building materials. Two major recharges of wood can be identified, each associated with a distinct peak in surface temperature. The living room is heated by an electric radiator. During periods without stove use, the radiator operates with an on-off control, with power varying between zero and its maximum level. The radiator switches off when the indoor temperature exceeds approximately 23 ${}^{\circ }$C, which appears to be its set point, and switches on again when the temperature drops below this threshold. The total household electric power varies between roughly zero and 6 kW.

The effect of stove operation is clearly visible. The decrease in electric power consumption is about 1 kW, corresponding to the radiator power that is offset by the heat delivered from the stove. This value is well below the nominal 4 kW capacity of the stove. However, the indoor temperature remains elevated long after the stove has stopped operating. As a result, the number of on-off cycles of the electric radiator is reduced compared to the period before stove operation. This indicates that the influence of the stove on electrical power demand extends over a long period and reduces the average space heating load.

## 4 Experimental Design, Materials and Methods

**Fig. 6:**
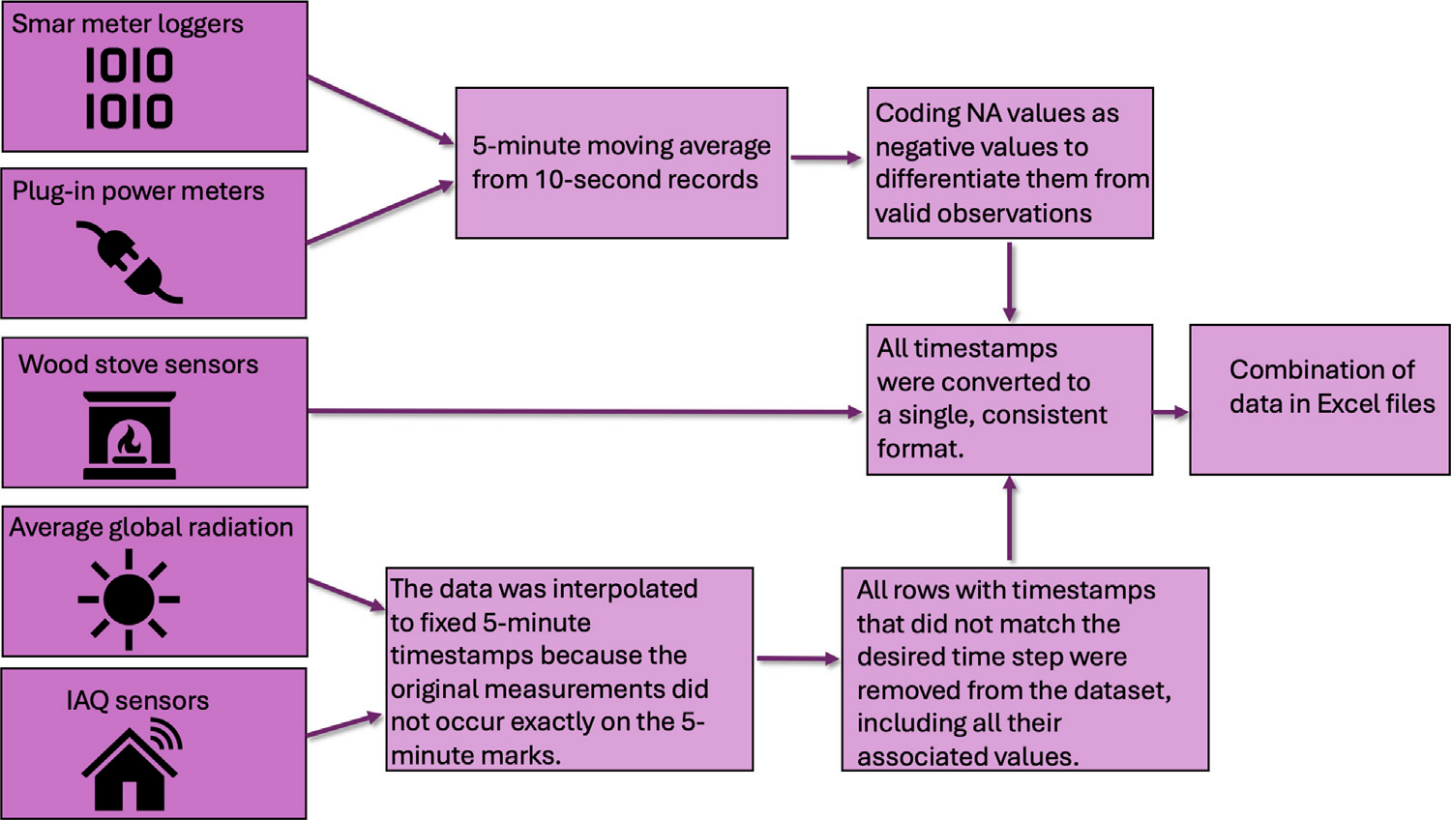
Schematic workflow of the cleaning process, illustrating the sequence of steps involved and their interconnections.

Before presenting the sensors and experimental design, it should be noted that the cleaned dataset has a temporal resolution of 5 min. For electric power measurements, each value represents a sliding average over the preceding 5 min, whereas for all other variables, the values correspond directly to the sensor readings obtained at 5-min intervals. Fig. 6 illustrates the schematic workflow for the cleaning process.

Due to interruptions during the experiment, missing data is present and has not been interpolated in the cleaned dataset. Several interruptions occurred during the pilot phase. In this phase, data port forwarding was used to monitor the data collected from the Raspberry Pi. Due to unstable internet connectivity in the home, substantial portions of the power data were missing. In the other two houses, this approach was replaced with cloud based monitoring. Although this method improved accessibility, it also experienced occasional data losses.

Another challenge was the high power consumption of the wood stove loggers in the pilot house, which depleted the batteries within a few days. This issue was later resolved by connecting the loggers to a power converter plugged into a wall outlet. In addition, the IAQ sensors were initially equipped with low quality batteries that discharged rapidly. Subsequently, these were replaced with high quality batteries to ensure stable operation.

Sensors and loggers were grouped into four categories, as described below. A summary of sensor types, measurement ranges, and accuracies is provided in Table 4.

**Table 4:** Sensors, measurement ranges, and accuracies.

Device	Type	Range	Accuracy
IAQ sensor	Temperature	N/A	±1 ${}^{\circ }$C
	CO_2_ (Non-Dispersive Infra-Red)	400∼5000 ppm	±30 ppm ±3 %
			within 15–35 ${}^{\circ }$C
			& 0–80 % RH
	RH	N/A	±3 %
	Pressure	N/A	±0.15 hPa
	TVOC (Metal Oxide MEMS)	0∼10,000 ppb	N/A
	Radon (Passive Diffusion Chamber)	0∼500 pCi/L	N/A
	PM_1_ (Laser Scattering Based Optical Particle Counter)	N/A	N/A
	PM_2.5_ (Laser Scattering Based Optical Particle Counter)	0∼1000 μg/$m^{3}$	± (5 μg/$m^{3}$+20 %)
Wood stove	Temperature sensor inside device	−20∼60 ${}^{\circ }$C	±0.3 ${}^{\circ }$C
sensors	RH	10∼90 %	±3 %
	Ambient light	0∼83k lux	±2 %
	External temperature sensor on stove surface (PT100)	−200∼600 ${}^{\circ }$C	±(2 %+1 ${}^{\circ }$C)

### 4.1 IAQ Sensors

Two types of IAQ sensors were used. The first type measured radon, TVOC, CO_2_, relative humidity, temperature, and air pressure, while the second type measured the same parameters in addition to PM_1_ and PM_2.5_. The temperature sensor in the IAQ devices measures the air temperature. Although mean radiant temperature is an important variable, particularly for thermal comfort assessment in buildings, it is more challenging to measure during long-term campaigns in occupied dwellings, for example, when using a black globe thermometer. The sensors were connected to Wi-Fi and transmitted data to the cloud at 5-min intervals. They were battery powered devices operating on AA batteries. The TVOC and CO_2_ readings required a settlement time of approximately seven days.

### 4.2 Average global radiation on a horizontal plane

The average global radiation on a horizontal plane was obtained for the Trondheim–Gløshaugen station (SN68173) from the Norwegian Climate Service Centre^1^ and is represented as G (W/$m^{2}$) in the datasets. No information was available regarding the data collection methods, sensor types, or sensor accuracy.

### 4.3 Plug-in power meters

The power consumption of the electric heating panels was monitored using Zigbee smart mini plugs with an accuracy of ±2%. Negative values in the raw data indicate missing entries (NA) that were intentionally encoded as negative numbers within the measurement platform to differentiate them from valid observations.

### 4.4 Wood stove sensors

A PT100 probe was used to measure the external surface temperature of the wood stove. It was mounted directly on the stove’s external surface. This probe was connected to a data logger that transmitted the measurements to the cloud via Wi-Fi. The same logger also included internal sensors to measure air temperature, relative humidity, and ambient light, and all data was recorded at a resolution of 5-min.

### 4.5 Smart meter logger

The smart meter logger was connected to the HAN port of the smart meter (AMS) and was capable of collecting data every 2 s; however, due to limitations of our monitoring platform, the data were recorded at 10-s intervals. Both smart plugs and smart meter logger communicated through a Zigbee gateway linked to a Raspberry Pi 3, with a data resolution of 10 s. If negative values appear in the raw data, they represent missing values (NA) that were intentionally set to negative numbers in the measurement platform to distinguish them from valid measurements.

## Ethics Statements

All datasets were provided with consent from the building owners.

## Declaration of Generative AI and AI-Assisted Technologies in the Writing Process

Generative AI and AI-assisted technologies have been used to improve the language of this paper.

## CRediT authorship contribution statement

**Abolfazl Mohammadabadi:** Conceptualization, Methodology, Software, Validation, Formal analysis, Writing – original draft. **Øyvind Skreiberg:** Writing – review & editing, Supervision. **Laurent Georges:** Conceptualization, Methodology, Validation, Formal analysis, Writing – original draft, Writing – review & editing, Supervision.

## Data Availability

Mendeley DataDetailed Operational Data for Three Norwegian Houses Equipped with Wood Stoves: Sub-Hourly Room-Level Measurements of Indoor Environmental Quality, Space-Heating Power, Stove Surface Temperature, and.

## Footnotes

1 https://seklima.met.no/.

## References

[1] Sartori I., Walnum H.T., Skeie K.S., Georges L., Knudsen M.D., Bacher P., Candanedo J., Sigounis A.-M., Prakash A.K., Pritoni M. (2023). Sub-hourly measurement datasets from 6 real buildings: energy use and indoor climate. Data Brief.

[2] Melgaard S.P., Johra H., Nyborg V.Ø., Marszal-Pomianowska A., Jensen R.L., Kantas C., Larsen O.K., Hu Y., Frandsen K.M., Larsen T.S. (2024). Detailed operational building data for six office rooms in denmark: occupancy, indoor environment, heating, ventilation, lighting and room control monitoring with sub-hourly temporal resolution. Data Brief.

[3] Bacher P., Madsen H. (2011). Identifying suitable models for the heat dynamics of buildings. Energy Build..

[4] Yu X., Skeie K.S., Knudsen M.D., Ren Z., Imsland L., Georges L. (2022). Influence of data pre-processing and sensor dynamics on grey-box models for space-heating: analysis using field measurements. Build. Environ..

[5] Thorsteinsson S., Kalaee A.A.S., Vogler-Finck P., Stærmose H.L., Katic I., Bendtsen J.D. (2023). Long-term experimental study of price responsive predictive control in a real occupied single-family house with heat pump. Appl. Energy.

[6] Lien S.K., Canaydin A., Miller C., Fu C., Kazmi H., Rajasekharan J. (2025). Cross-domain disaggregation of electricity for heating in all-electric school buildings–learning from school buildings with district heating. Energy Build..

[7] Mohammadabadi A., Skreiberg Ø., Georges L. (2023). Energy Informatics Academy Conference.

